# New species and records of Hydroptilidae (Trichoptera) from Venezuela

**DOI:** 10.3897/zookeys.185.2909

**Published:** 2012-04-23

**Authors:** Robin E. Thomson, Ralph W. Holzenthal

**Affiliations:** 1 Department of Entomology, University of Minnesota, 219 Hodson Hall, 1980 Folwell Avenue, St. Paul, Minnesota, 55108, USA; 2 Department of Entomology, National Museum of Natural History, Smithsonian Institution, Washington, DC 20013, USA

**Keywords:** Trichoptera, caddisflies, Hydroptilidae, *Anchitrichia*, *Hydroptila*, *Metrichia*, *Neotrichia*, *Ochrotrichia*, *Oxyethira*, *Rhyacopsyche*, new species, Neotropical

## Abstract

Eight new species of Hydroptilidae (Trichoptera) from Venezuela are described: *Acostatrichia digitata* **sp. n.**, *Hydroptila cressae* **sp. n.**, *Metrichia botrychion* **sp. n.**, *Ochrotrichia spira* **sp. n.**, *Oxyethira bettyae* **sp. n.**, *Oxyethira quiramae* **sp. n.**, *Oxyethira redunca* **sp. n.**, and *Rhyacopsyche shorti* **sp. n.**New country records for Venezuela of 2 additional species, *Neotrichia feolai* Santos & Nessimian, 2009 and *Oxyethira picita* Harris & Davenport, 1999, are also provided. Illustrations of male genitalia are provided with each description.

## Introduction

Caddisflies, or Trichoptera, are a diverse order of insects with ~15,000 described species and 100s of new species awaiting description (Holzenthal et al. 2011). Trichoptera faunal diversity is particularly impressive in the Neotropical biogeographical region, where recent inventories have revealed up to 75% of collected species to be undescribed (Holzenthal et al. 2007) Hydroptilidae is the largest family in the order, including 75 genera, ~2,000 described species found all over the world, and a high number of undescribed species. As their common name, microcaddisflies, suggests, hydroptilids are minute with few larger than 5 mm. The aquatic larvae construct portable or fixed silken purse-like cases in the final instar (Wiggins 2004). The larvae of many species feed on algae, while some feed on moss microphylls (Wiggins 2004; Carins and Wells 2008). Some have been known to be predatory, while others are parasitoids (Wells 1985, 2005).

In this paper, we describe 8 new hydroptilid species in 6 genera from Venezuela.We also provide new country records for Venezuela for 2 species, *Neotrichia feolai* Santos & Nessimian, 2009 and *Oxyethira picita* Harris & Davenport, 1999. The material was collected as part of a project under the direction of Dr. Andrew Short, University of Kansas, USA, to inventory the aquatic Coleoptera and other aquatic insect orders of Venezuela. In June, 2010, a team of 4 American and 4 Venezuelan entomologists collected aquatic insects in the southern half of Venezuela, including the *llanos* of Guárico state, the southern tributaries of the upper to middle Orinoco River basin, and the *Gran Sabana* of Bolívar state, and, in northern Venezuela, the Turimiquire Mountains of Monagas state. About 90 species of Trichoptera were collected, including about 25 new species of which the new Hydroptilidae are described here.

## Materials and methods

Morphological terminology used for male genitalia of specimens in the genus *Oxyethira* follows that of Kelley (1984), for the genus *Metrichia* that of Flint (1972), and for the genus *Rhyacopsyche* that of Wasmund and Holzenthal (2007). All others follow the terminology of Marshall (1979). For simplicity, paired structures are discussed in the singular. Procedures for specimen preparation followed those explained in detail by Blahnik et al. (2007). For specimen examination and illustration, cleared genitalia were placed in a watch glass with glycerin and small glass beads. The glass beads held the genitalia in place and allowed structures to be viewed in precise lateral, dorsal, and ventral positions. Genitalia were examined with an Olympus BX41 compound microscope at 250–500 × magnification. Structures were traced in pencil with the use of a *camera lucida* (drawing tube) mounted on the microscope. Pencil sketches were then scanned (Fujitsu ScanSnap S1500M scanner), edited in Adobe Photoshop (v. 9.0.2, Adobe Systems Inc.), and used as a template in Adobe Illustrator (v. 13.0.2, Adobe Systems Inc.) to be digitally inked. Electronic “drawing” was completed with the aid of a graphics tablet (Bamboo Fun, Wacom Company, Limited). Species descriptions were constructed using the program DELTA (Dallwitz et al. 1999) and specimen management followed the procedures outlined by Holzenthal and Andersen (2004). Each pinned specimen examined during the study was affixed with a barcode label (4 mil polyester, 8 × 14 mm, code 49) bearing a unique alphanumeric sequence beginning with the prefix UMSP. Specimens in alcohol were given a single barcode label to represent all those in a single vial. The prefix is not meant to imply ownership by the University of Minnesota Insect Collection (UMSP), but only to indicate that the specimen was databased at that collection. Types of species described and other material examined are deposited in the University of Minnesota Insect Collection, St. Paul, Minnesota (UMSP), the National Museum of Natural History, Smithsonian Institution, Washington, D.C. (NMNH), and the Museo del Instituto de Zoología Agrícola, Maracay, Venezuela (MIZA).

## Taxonomy

### Acostatrichia digitata

Thomson & Holzenthal sp. n.

urn:lsid:zoobank.org:act:85981ECC-3397-4968-ACBC-B6EE2098DA5B

http://species-id.net/wiki/Acostatrichia_digitata

Fig. 1

#### Diagnosis.

This species is most similar to *Acostatrichia fimbriata* Flint, 1974 but can be distinguished by a mesoventral process on abdominal segment VII with an apex that is truncate and rugose, not pointed. The posterolateral process of abdominal segment VIII bears digitate projections apically, unlike the spines on *Acostatrichia fimbriata*. Additionally, the subgenital appendage of *Acostatrichia digitatus* is pointed apically instead of rounded.

#### Description.

**Figure 1.**
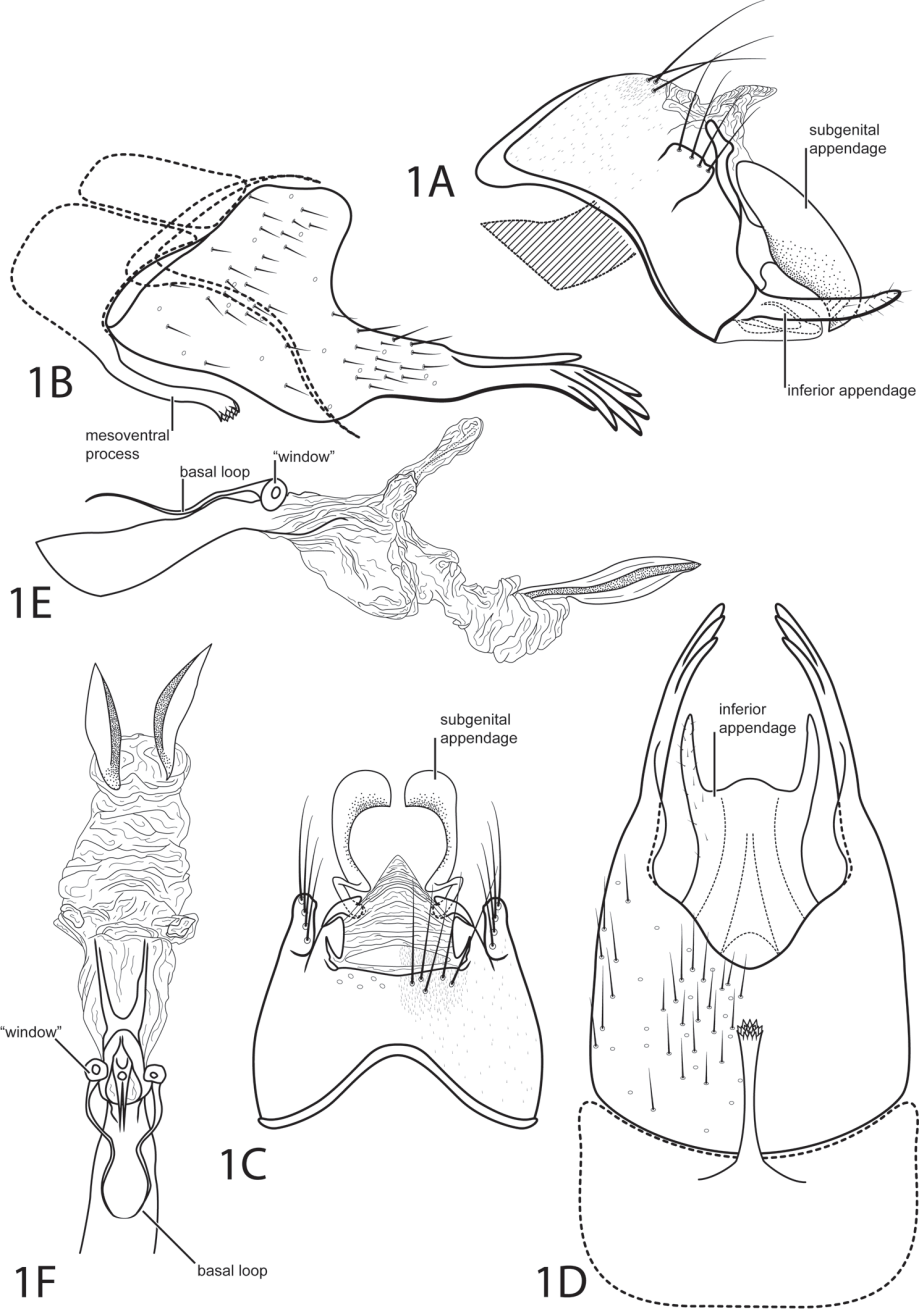
*Acostatrichia digitata* sp. n. Male genitalia: **A** segments IX–X, lateral (base of phallus crosshatched) **B** segments VII–VIII and segment IX anterolateral margin, lateral **C** segments IX–X, dorsal **D** segments VII–IX, ventral **E** phallus, lateral **F** phallus, dorsal.

*Male*. Length of forewing 2.7 mm (n=1). Head unmodified, with 3 ocelli; antennae unmodified. Tibial spur count 1, 3, 4. Dorsum of head dark brown with pale yellow setae; thorax dark brown with pale yellow setae dorsally, light brown ventrally; leg segments with light brown setae. Forewings covered with fine yellow setae and small scattered patches of dark brown setae. *Genitalia*. Abdominal sternum VII with long mesoventral process, apex truncate, rugose. Segment VIII anterolateral margin straight, posterolateral margin greatly elongate into narrow structure bearing digitate apical projections; ventrally posterior margin concave, mildly crenulated. Segment IX anterolateral margin acute, posterolateral margin broadly convex; with mesolateral quadrate structure bearing prominent setae (see Fig. 1A); dorsally with posterior margin straight. Subgenital appendage paired, broadly rounded with apicoventral point, dorsally with rounded emargination on inner edge. Inferior appendage setose, narrow, rod-like, fused latero-ventrally with subgenital appendage, in ventral view with semiquadrate apical emargination (see Fig. 1D). Tergum X membranous, triangular in dorsal view. Phallus tubular basally with median complex bearing basal loop and pair of circular “windows”, apex with pair of elliptic plates, strongly sclerotized mesolaterally.

#### Material examined.

**Holotype male:** **VENEZUELA: Bolívar:** E Tumeremo, W Bochinche, Río Botonamo, 07°25.462'N, 61°14.318'W, 150 m, 13.vii.2010, UV light, Holzenthal, Thomson, Cressa (UMSP000095201) (UMSP).

#### Etymology.

The Latin word *digitatus* meaning “having fingers”,referring to the digitate projections on the posterolateral process of the VIIIth segment.

### Hydroptila cressae

Thomson & Holzenthal sp. n.

urn:lsid:zoobank.org:act:22765A69-4EE1-4DA6-BB78-E100D0C6EF33

http://species-id.net/wiki/Hydroptila_cressae

Fig. 2

#### Diagnosis.

This species is most similar to *Hydroptila denza* Ross, 1948, but differs in the shape of the projection on the posterolateral margin of abdominal segment IX. This projection is more pointed and is curved downward, or decurved, in *Hydroptila cressae*, while it is straight and more blunt in *Hydroptila denza*. The triangular subgenital process seen in *Hydroptila denza* is not apparent in *Hydroptila cressae*.Additionally, tergum X of *Hydroptila cressae* contains an internal apodeme that is not apparent in *Hydroptila denza*.

#### Description.

**Figure 2.**
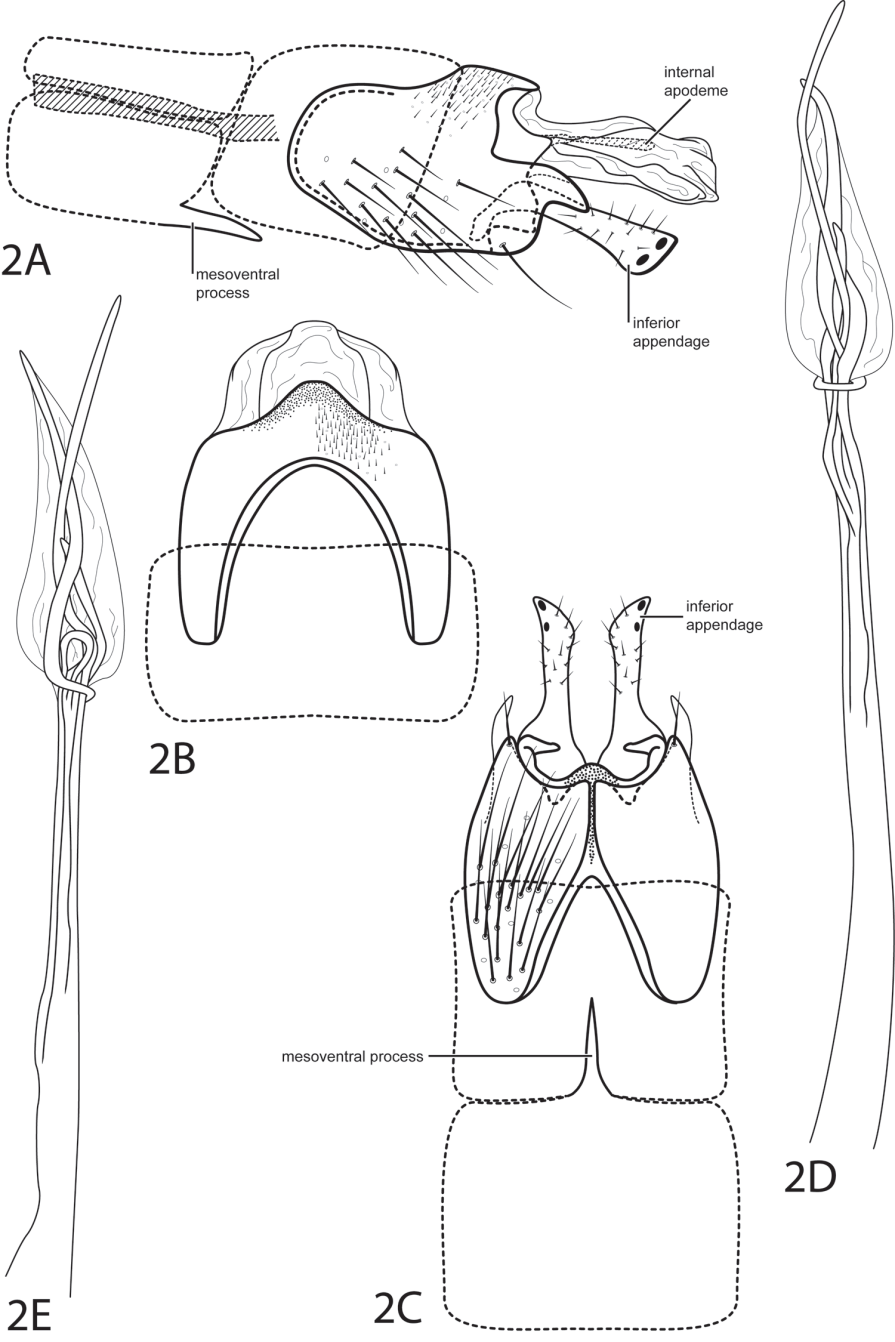
*Hydroptila cressae* sp. n. Male genitalia: **A** segments VII–X, lateral (base of phallus crosshatched) **B** segments VIII-X, dorsal **C** segments VII–IX, ventral **D** phallus, lateral **E** phallus, dorsal.

*Male*. Length of forewing 2.0 mm (n=1). Head unmodified, without ocelli; antennae unmodified. Tibial spur count 0, 2, 4. Dorsum of head brown with pale yellow setae; thorax brown with light brown setae dorsally, light brown ventrally; leg segments with light brown setae. Forewings covered with fine light brown setae with small dark brown patch of setae at apex. *Genitalia*. Abdominal sternum VII with simple, slender, pointed mesoventral process. Segment VIII unmodified. Segment IX anterolateral margin convex, posterolateral margin with pointed projection, curving slightly ventrad; dorsally with posterior margin convex. Inferior appendage setose, with narrow base, apex truncate with pair of dark points on internal face. Tergum X membranous, extending past inferior appendage, containing internal sclerotized apodeme (see Fig. 2A). Phallus narrow, elongate; apex membranous, ovate, with elongate, slender spines extending past membranous region.

#### Material examined.

**Holotypemale:** **VENEZUELA: Bolívar:** Gran Sabana, E. Pauji, “Río Curvita”, 04°31.237'N, 61°31.591'W, 869 m, 15–16.vii.2010, UV light, Holzenthal, Thomson, Cressa (UMSP000095196) (UMSP).

#### Etymology.

Named in honor of Dr. Claudia Cressa, an aquatic ecologist at the Universidad Central de Venezuela and friend and colleague of the authors.

### Metrichia bostrychion

Thomson & Holzenthal sp. n.

urn:lsid:zoobank.org:act:13F7C864-63DB-4360-92FA-D3CB3331DBA3

http://species-id.net/wiki/Metrichia_bostrychion

Fig. 3

#### Diagnosis.

This species is most similar to *Metrichia anisoscola* (Flint, 1991), but differs in the shape of the inferior appendage, which is less elongate in *Metrichia bostrychion* and more suborbicular. The dorsolateral hook in *Metrichia bostrychion* is also stouter than that of *Metrichia anisoscola*. *Metrichia bostrychion* can also be distinguished by the 3rd spine on the phallus which spirals dorsally.

#### Description.

**Figure 3.**
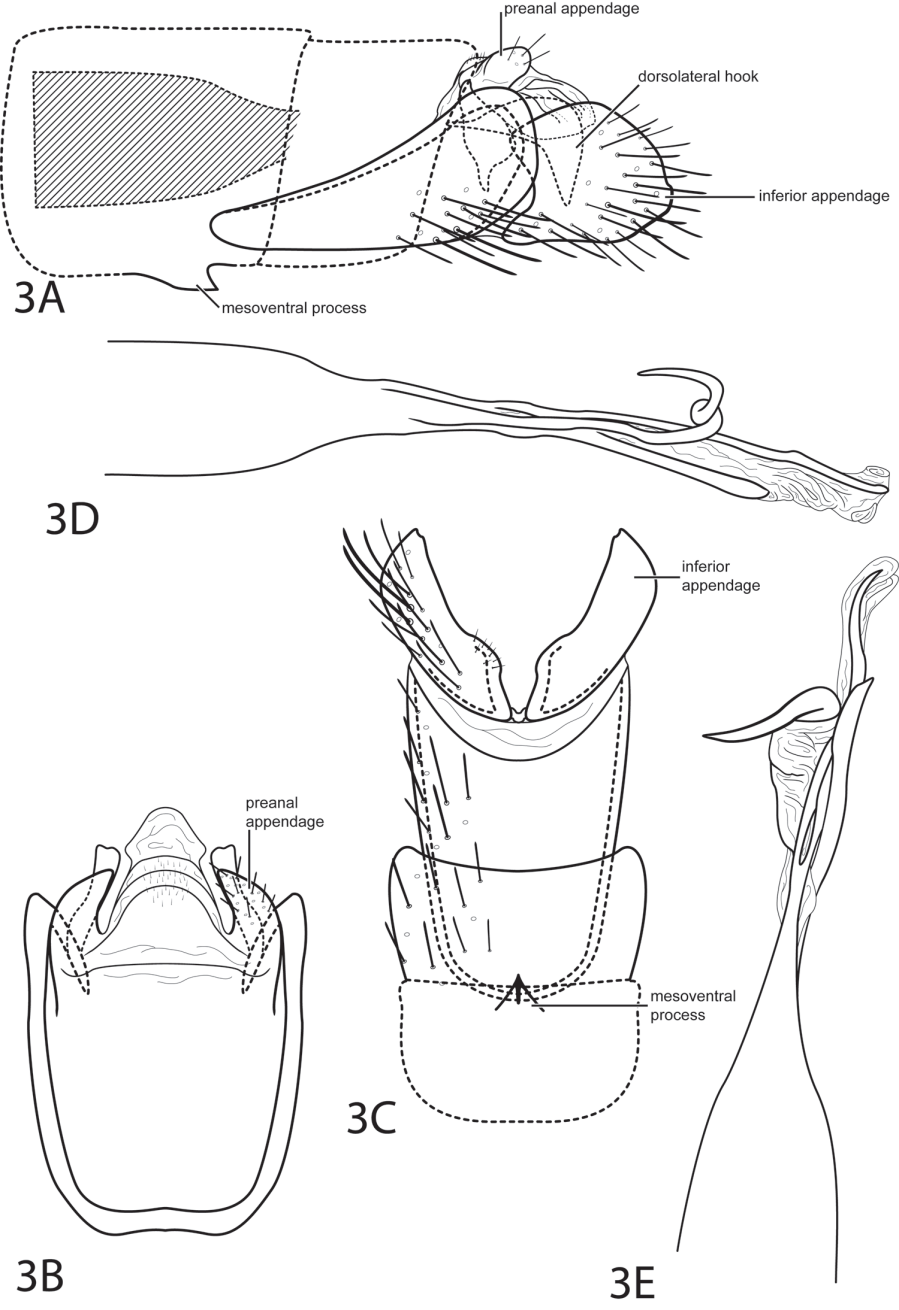
*Metrichia bostrychion* sp. n. Male genitalia: **A** segments VII–X, lateral (base of phallus crosshatched) **B** segments IX–X, dorsal **C** segments VII–IX, ventral **D** phallus, lateral **E** phallus, dorsal.

*Male*. Length of forewing 1.8 mm (n=1). Head unmodified, with 3 ocelli; antennae unmodified. Tibial spur count 1, 3, 4. Dorsum of head dark brown with white setae; thorax dark brown with white and dark brown setae dorsally, brown ventrally; leg segments with brown setae. Forewings covered with fine dark brown setae with small patch of light brown setae at apex. Abdomen with internal sacs between segments IV–V. Dorsolateral setal brushes on segments IV and V. *Genitalia*. Abdominal sternum VII with short, pointed mesoventral process. Segment VIII unmodified. Segment IX anterolateral margin very elongate, narrowing, withdrawn into segments VII–VIII, posterolateral margin convex; dorsally with posterior margin membranous, flat. Preanal appendage (cercus) short, rounded. Dorsolateral hook stout, strongly decurved (see Fig. 3A). Inferior appendage suborbicular with shallow posterolateral emargination, extends as high as segment IX. Tergum X membranous, apex subdeltoid in dorsal view. Phallus widest at base, narrowing to median constriction, membranous apex with 3 spines, 1st and 3rd slender, elongate, 2nd spiraling dorsad.

#### Material examined.

**Holotype male:** **VENEZUELA: Monagas:** Guachero Cave National Park at La Paila waterfall, 10°10.322'N, 63°33.315'W, 1110 m, 20–21.vii.2010, sweep net, Holzenthal, Thomson (UMSP000095197) (UMSP).

#### Paratype.

same data as holotype, 1 female (UMSP).

#### Etymology.

The diminutive of the Greek word *bostrychos* meaning “curl”, referring to the small spiral in the second apical spine on the phallus.

### Neotrichia feolai

Santos & Nessimian, 2009 redescription and new country record

http://species-id.net/wiki/Neotrichia_feolai

Fig. 4

Neotrichia feolai Santos & Nessimian, 2009: 766 [Type locality: Brazil, Amazonas, Rio Preto da Eva (tributary to Rio Preto da Eva); INPA; male].

#### Diagnosis.

*Neotrichia feolai* was previously only known from the male holotype collected from Brazil, Amazonas. Eight males were collected for the first time from Venezuela, representing a new record in this study for the country. Original illustrations did not include the distinctive subgenital appendage, but specimens from Venezuela match all other characteristics of the Brazilian species perfectly. Some of our specimens are dry, while the holotype was collected in alcohol, allowing us to to describe coloration. We have also described and illustrated the subgenital plate not seen in the original illustration.

According to the original authors, this species is most similar to *Neotrichia biuncifera* Flint, 1974. The shapes and lengths of the bracteole and inferior appendage are similar, but *Neotrichia feolai* can be distinguished by having only a single spine at the apex of the phallus.

#### Redescription.

**Figure 4.**
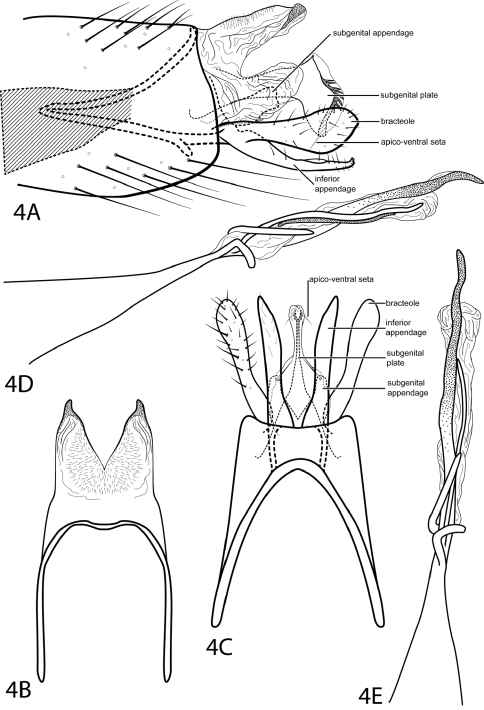
*Neotrichia feolai* Santos & Nessimian, 2009. Male genitalia: **A** segments VIII–X, lateral (base of phallus crosshatched) **B** segments IX–X, dorsal **C** segment IX, ventral **D** phallus, lateral **E** phallus, dorsal.

*Male*. Length of forewing 1.6–1.9 mm (n=8). Head unmodified, with 3 ocelli; antennae unmodified. Tibial spur count 0, 2, 3. Dorsum of head brown with light brown setae; thorax brown with light brown setae dorsally, light brown ventrally; leg segments with light brown setae. Forewings covered with fine light brown setae with small patches of dark brown setae. *Genitalia*. Abdominal sternum VII without mesoventral process. Segment VIII unmodified. Segment IX anterolateral margin strongly narrowing, withdrawn into segment VIII, posterolateral margin fused dorsally with tergum X (see Fig. 4B). Subgenital plate fused, diamond-shaped with pair of apico-ventral setae, posterior margin bearing paired row of sclerotized spines within membranous layer (see Figs. 4A, 4C). Bracteole spatulate, extended evenly with inferior appendage. Inferior appendage setose, laterally narrow and rod-like, fused latero-ventrally with subgenital appendages, ventrally with semiquadrate apical emargination. Tergum X membranous, bearing minute dorsal setae, with deep emargination both laterally and dorsally, dorsal lobe with sclerotized apex. Phallus with wide tubular base narrowing to median constriction, membranous apex with spiral process and slender apical spines.

#### Material examined.

**VENEZUELA: Guárico:** Santa Rita, Morichal de los Becerros, 08°09.044’N, 62°35.149’W, 66 m, 6.vii.2010, UV light, Holzenthal, Thomson, 8 males (5 in alcohol) (UMSP, NMNH, MIZA).

### Ochrotrichia spira

Thomson & Holzenthal sp. n.

urn:lsid:zoobank.org:act:83832CD2-268B-4AA3-A5EC-F6FAB463D9F6

http://species-id.net/wiki/Ochrotrichia_spira

Fig. 5

#### Diagnosis.

This species is most similar to *Ochrotrichia raposa* Bueno-Soria & Santiago-Fragoso, 1992. Both have a simple, threadlike phallus and the large inferior appendage bears patches of black pegs on its internal face. The inferior appendage of *Ochrotrichia spiralis*, however, is parallel-sided with a truncate apex. Also, the sclerotized processes extending from tergum X of *Ochrotrichia spiralis* are easily distinguishable from those of *Ochrotrichia raposa*, in particular the large, spiral process.

#### Description.

**Figure 5.**
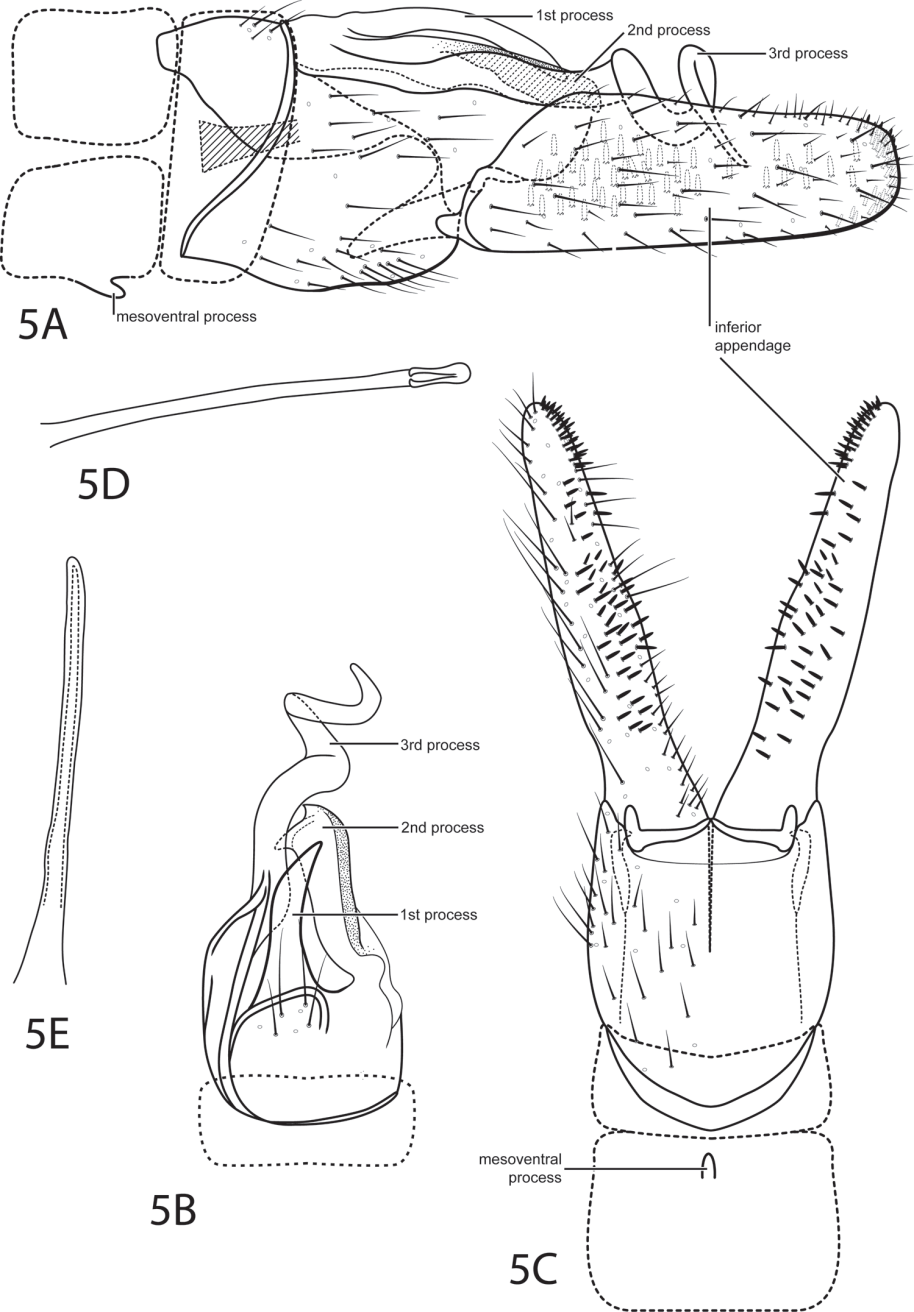
*Ochrotrichia spira* sp. n. Male genitalia: **A** segments VII-X, lateral (base of phallus crosshatched) **B** segments VIII–X, dorsal **C** segments VII–IX, ventral **D** phallus, lateral **E** phallus, dorsal.

*Male*. Length of forewing 2.6–2.7 mm (n=3). Head unmodified, with 3 ocelli; antennae unmodified. Tibial spur count 0, 3, 4. Dorsum of head dark brown with pale yellow setae; thorax light brown with light brown setae dorsally, light brown ventrally; leg segments with light brown setae and patches of dark brown. Forewings covered with fine brown setae with small patches of dark brown setae near apex. *Genitalia*. Abdominal sternum VII with short, rounded mesoventral process. Segment VIII unmodified. Segment IX anterolateral margin concave, posterolateral margin fused dorsally with tergum X. Inferior appendage setose, 3 times longer than wide, parallel-sided, apex truncate, inner surface bearing many short, stout spines distributed as in Figs. 5A, 5C. Tergum X sclerotized, highly developed with 3 processes: 1st simple, slender pointed; 2nd with heavily sclerotized edge, large subapical point, small apical point; 3rd strongly spiraled, extended past other processes. Phallus tubular, elongate, threadlike.

#### Material examined.

**Holotypemale:** **VENEZUELA: Monagas:** Guachero Cave National Park, 10°10.322'N, 63°33.315'W, 1110 m, 20–21.vii.2010, UV light, Holzenthal, Thomson, Cressa (UMSP000095193) (UMSP).

#### Paratypes.

same data as holotype, 2 males (UMSP, NMNH).

#### Etymology.

The Latin word *spiralis* meaing “spiral”, referring to the strongly spiraled process extending from tergum X.

### Oxyethira (Tanytrichia) bettyae

Thomson & Holzenthal sp. n.

urn:lsid:zoobank.org:act:9B85F4D2-070A-4192-BD8D-E4DF432D19AA

http://species-id.net/wiki/Oxyethira_bettyae

Fig. 6

#### Diagnosis.

This species is placed in the subgenus *Tanytrichia* according to the characters given by Kelley (1985): segment VIII venter excised to anterior margin, segment IX elongate and extending into segment VI, the absence of segment IX dorsum, and a phallus bearing two long lateral processes originating at the midlength. This species is most similar to *Oxyethira longissima* Flint, 1974. The phallus is very similar, bearing long paired processes sharply bent back anteriorly. However, the subgenital process of *Oxyethira longissima* is more strongly arched and much more slender in lateral view than that of *Oxyethira bettyae*. Also, when viewed ventrally, the bilobed process of *Oxyethira bettyae* is wider basally than *Oxyethira longissima*.

#### Description.

**Figure 6.**
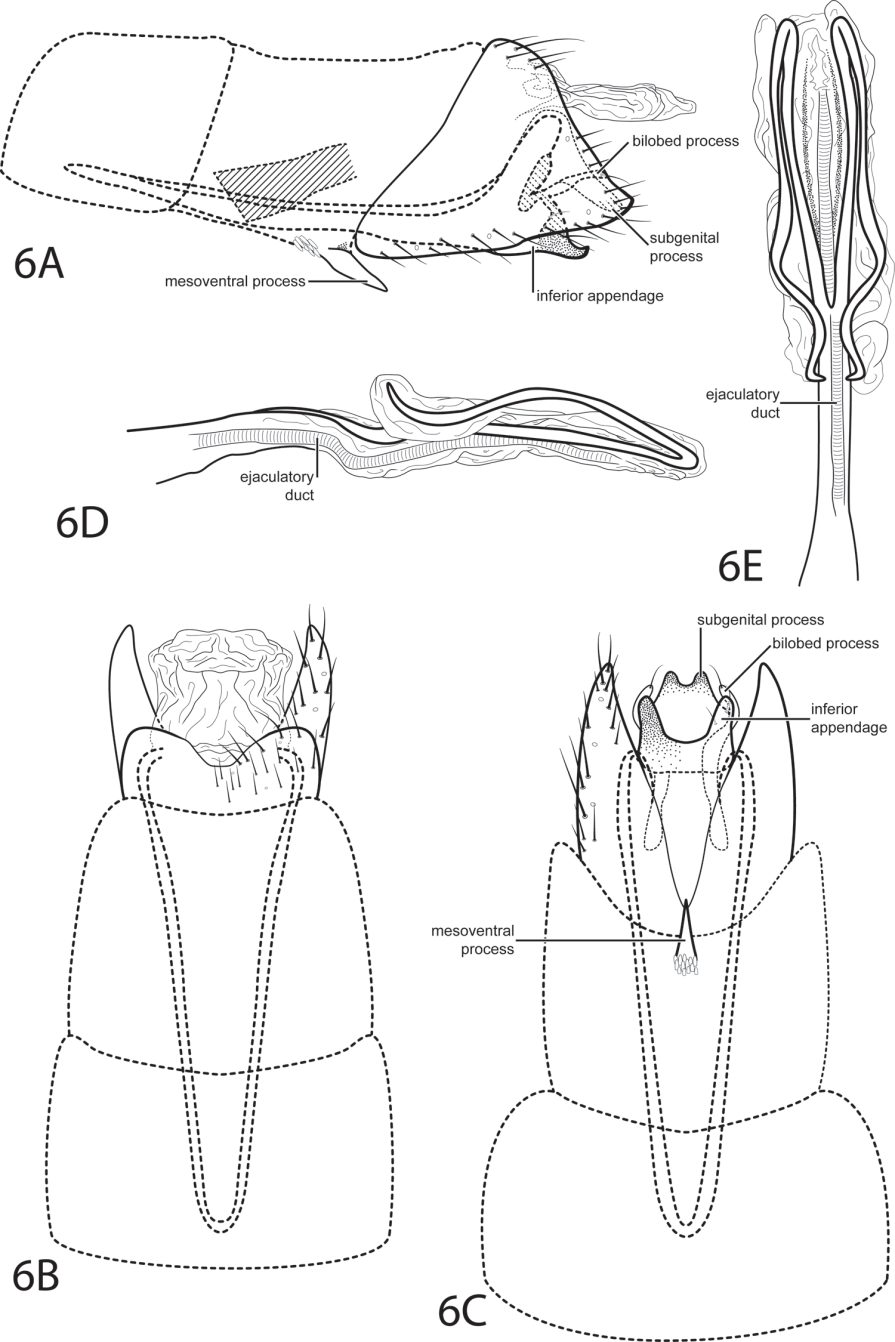
*Oxyethira bettyae* sp. n. Male genitalia: **A** segments VI–X, lateral (base of phallus crosshatched) **B** segments VI–X, dorsal **C** segments VI–IX, ventral **D** phallus, lateral **E** phallus, dorsal.

*Male*. Length of forewing 2.0–2.2 mm (n=6). Head unmodified, with 3 ocelli; antennae unmodified. Tibial spur count 0, 3, 4. Dorsum of head dark brown with pale yellow setae; thorax brown with light brown setae dorsally; leg segments with light brown setae. Forewings covered with fine light brown setae and small scattered patches of dark brown setae and golden brown setae. *Genitalia*. Abdominal sternum VII with simple, pointed mesoventral process with small patch of stout pegs basally. Segment VIII anterolateral margin straight, posterolateral margin pointed; dorsally posterior margin with rounded emargination; ventrally posterior margin with deeply divided. Segment IX anterolateral margin very narrow and very elongate, withdrawn into segments VI–VIII, posterolateral margin straight, not extended posteriorly past segment VIII. Subgenital process fused, apex with small rounded emargination (see Fig. 6C). Bilobed process slender, extending posteriad. Inferior appendage fused with deep apical emargination, sparsely setose, heavily sclerotized, apex acute, upturned in lateral view. Tergum X membranous, quadrate dorsally, oblong ventrally. Phallus with tubular basal half, apical half membranous; 2 long, lateral processes beginning at midlength, very sharply curved backward, or recurved.

#### Material examined.

**Holotypemale:** **VENEZUELA: Guárico:** UCV San Nicolasito Field Station, 08°8.296'N, 66°24.459'W, 62 m, 5.vii.2010, UV light, Holzenthal, Thomson (UMSP000095178) (UMSP).

#### Paratypes.

same data as holotype, 1 male (UMSP); **Venezuela: Bolívar:** Los Pijiguaos at rock outcrop, 6°35.617'N, 66°49.238'W, 80 m, 7–8.vii.2010, UV light trap, Holzenthal, Thomson, 5 males (in alcohol) (UMSP, NMNH, MIZA).

#### Etymology.

Named in honor of the first author’s grandmother, Betty Welter, who passed away while this work was in progress.

### Oxyethira picita

Harris & Davenport, 1999 new country record

http://species-id.net/wiki/Oxyethira_picita

Oxyethira picita Harris & Davenport, 1999: 35 [Type locality: Peru, Loreto, edge of Rio Sucusari backwater, adjoining Explorama lodge; NMNH; male]. - Santos et al. 2009: 36, 43 [distribution].

#### Diagnosis.

*Oxyethira picita* was previously known only from the male holotype collected from Peru, Loreto. A single male was collected at a later date from Brazil, Amazonas (Santos et al. 2009). Five males were now collected for the first time from Venezuela, representing a new record for the country. All five males were collected in alcohol.

*Oxyethira picita* was placed in the subgenus *Tanytrichia* by Harris & Davenport (1999), although it was suggested that it also displayed some similarity to the subgenus *Loxotrichia*. The original description and illustration of *Oxyethira picita* are detailed and well done; further description or illustration was not thought necessary.

#### Material examined.

**VENEZUELA: Bolívar:** Campamento Río Aro, 07°37.443'N, 64°08.324'W, 90 m, 10–11.vii.2010, UV light, Holzenthal, Thomson, 4 males (in alcohol) (UMSP, MIZA). **VENEZUELA: Bolívar:** 30 km S Upata, roadside marsh, 07°22.239'N, 61°44.233'W, 163 m, 12.viii.2010, Short, Tellez, Camacho, 1 male (in alcohol) (NMNH).

### Oxyethira (Dactylotrichia) quiramae

Thomson & Holzenthal sp. n.

urn:lsid:zoobank.org:act:6C447B87-EA39-4D06-A28A-0702345D2930

http://species-id.net/wiki/Oxyethira_quiramae

Fig. 7

#### Diagnosis.

This species is placed in the subgenus *Dactylotrichia* according to the characters given by Kelley (1985): segment VIII venter excised nearly to the anterior margin and segment IX venter extending anteriorly through segments VIII–VI. This species is most similar to *Oxyethira hozosa* Harris & Davenport, 1999. Both species have short, blunt, ventrally triangular inferior appendages and a phallus with a distal curved process and an ejaculatory duct enclosed within the membranous apex. *Oxyethira quiramae* can be distinguished by a subgenital plate that is not as strongly decurved and lacks an acute apex in lateral view. Also, in *Oxyethira quiramae*, segment IX extends anteriorly past the posterior margin of abdominal segment VI.

#### Description.

**Figure 7.**
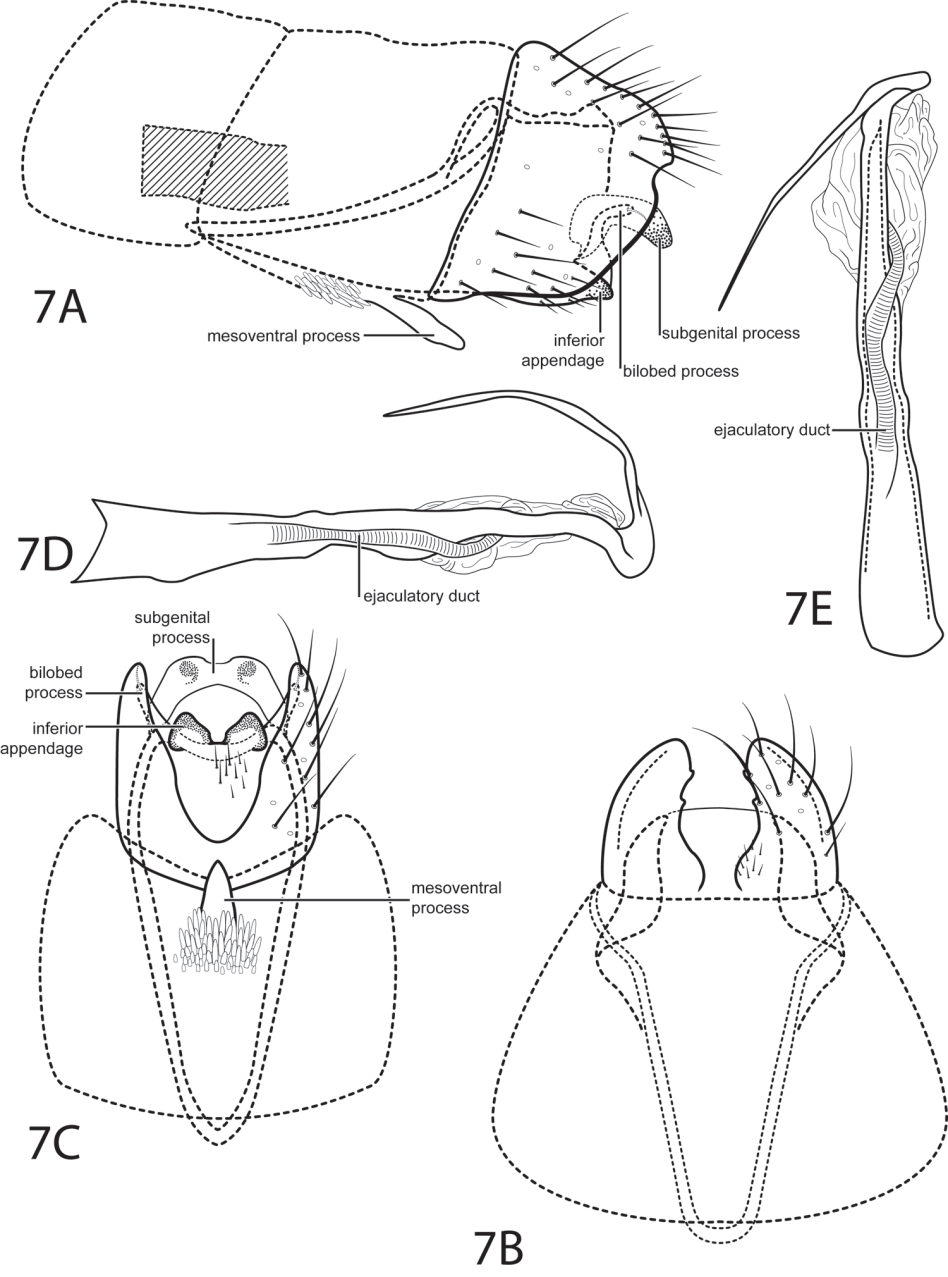
*Oxyethira quiramae* sp. n. Male genitalia: **A** segments VI–IX, lateral (base of phallus crosshatched) **B** segments VII–IX, dorsal **C** segments VII–IX, ventral **D** phallus, lateral **E** phallus, dorsal.

*Male*. Length of forewing 1.8–1.9 mm (n=3). Head unmodified, with 3 ocelli; antennae unmodified. Tibial spur count 0, 3, 4. Dorsum of head brown with light brown setae; thorax brown with light brown setae dorsally, light brown ventrally; leg segments with light brown setae. Forewings covered with fine brown setae with small scattered patches of light brown setae and small patches of dark brown setae near margins and apex. *Genitalia*. Abdominal sternum VII with simple, digitate mesoventral process with large patch of stout pegs basally. Segment VIII anterolateral margin straight, posterolateral margin convex with small mesal emargination; dorsally posterior margin with rounded emargination; ventrally deeply excised. Segment IX anterolateral margin narrow and elongate, withdrawn into segments VI–VIII, posterolateral margin straight, not extended posteriorly past segment VIII. Subgenital process fused distomesally, apex with shallow emargination, curving ventrad (see Fig. 7C). Bilobed process slender, curved, not extending posteriorly past segment VIII. Inferior appendage reduced, triangular, heavily sclerotized (see Fig. 7C). Tergum X not apparent. Phallus with tubular basal half, apical half membranous; apex elongate, slender, pointed, curving dorsad and sharply recurved.

#### Material examined.

**Holotype male:** **VENEZUELA: Guárico:** UCV San Nicolasito Field Station, 08°8.296'N, 66°24.459'W, 62 m, 5.vii.2010, UV light, Holzenthal, Thomson (UMSP000095179) (UMSP).

#### Paratypes.

same data as holotype, 2 males (NMNH, MIZA).

#### Etymology.

Named in honor of Gina Quiram, a friend and colleague of the first author, for all her help in the field.

### Oxyethira redunca

Thomson & Holzenthal sp. n.

urn:lsid:zoobank.org:act:C8A428D1-6558-479C-8AF8-8857D6E2A5FB

http://species-id.net/wiki/Oxyethira_redunca

Fig. 8

#### Diagnosis.

We have been unable to assign this species to a subgenus. The deep ventral excision of abdominal segment VIII and the extension of segment IX anteriorly into segment VII make it somewhat similar to *Loxotrichia*. However, the absence of a subgenital process precludes it from being placed with certainty in any of the current subgenera and distinguishes it from all other species.

#### Description.

**Figure 8.**
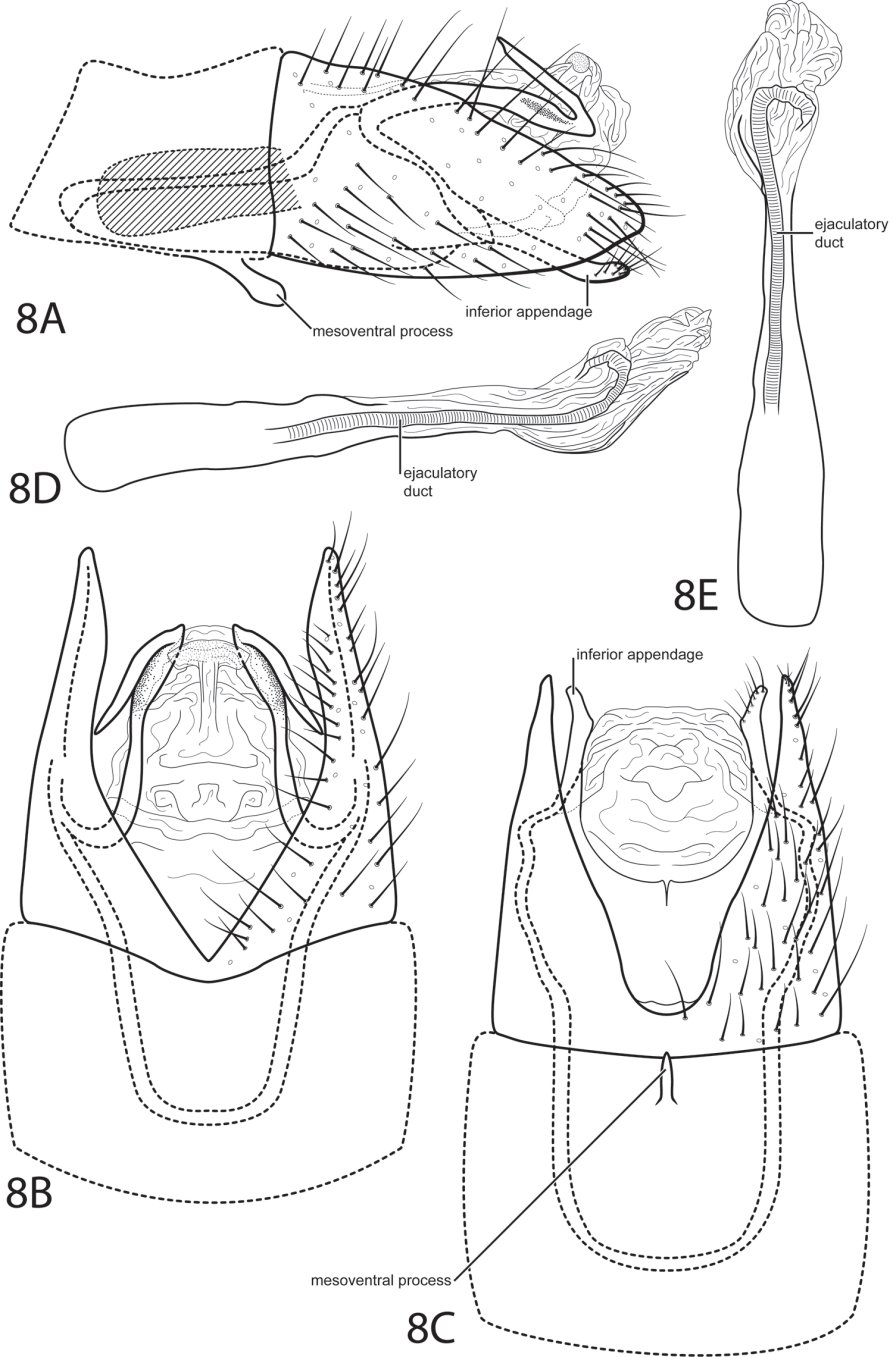
*Oxyethira redunca* sp. n. Male genitalia: **A** segments VII–X, lateral (base of phallus crosshatched) **B** segments VII–X, dorsal **C** segments VII–X, ventral **D** phallus, lateral **E** phallus, dorsal.

*Male*. Length of forewing 2.4 mm (n=1). Head unmodified, with 3 ocelli; antennae unmodified. Tibial spur count 0, 3, 4. Dorsum of head brown with pale yellow setae; thorax brown with pale yellow setae dorsally, pale yellow ventrally; leg segments with brown setae. Forewings covered with fine brown setae and elongate patches of light brown setae and small patches of dark brown setae near margins and apex. *Genitalia*. Abdominal sternum VII with spatulate mesoventral process. Segment VIII anterolateral margin straight, posterolateral margin acutely convex; dorsally posterior margin with deep acute emargination; ventrally posterior margin with deep rounded emargination. Segment IX anterolateral margin very elongate, narrowing, withdrawn into segments VII–VIII, posterolateral margin acute, not extending past segment VIII; dorsally bearing paired, elongate, slender processes, basal half extending posteriorly, apical half strongly bent anteriad. Subgenital process not apparent. Bilobed process not apparent. Inferior appendage setose, laterally narrow and rod-like, fused latero-ventrally with subgenital appendages, ventrally with semiquadrate apical emargination. Tergum X membranous, large, bearing elliptic patch of minute setae dorsally (see Fig. 8B). Phallus with tubular basal half, apical half membranous, convex ventrally, apex curving dorsad.

#### Material examined.

**Holotype male:** **VENEZUELA: Bolívar:** Gran Sabana, E. Pauji, “Río Curvita”, 4°31.237'N, 61°31.591'W, 869 m, 15–16.vii.2010, UV light, Holzenthal, Thomson, Cressa (UMSP000095176) (UMSP).

#### Etymology.

The Latin word *reduncus* meaning “bent backward”, referring to the sharply bent dorsal processes of segment IX.

### Rhyacopsyche shorti

Thomson & Holzenthal sp. n.

urn:lsid:zoobank.org:act:56EB1974-52AA-4EE1-9F95-357CD2AC3A4A

http://species-id.net/wiki/Rhyacopsyche_shorti

Fig. 9

#### Diagnosis.

This species is most similar to *Rhyacopsyche otarosa* Wasmund & Holzenthal, 2007. Both species display an inferior appendage with a bifid apex bearing a large rounded dorsal lobe. However, the ventral lobe is broadly pointed in *Rhyacopsyche shorti* and truncate in *Rhyacopsyche otarosa*. Additionally, when seen dorsally, the dorsolateral lobes of segment IX are rounded in *Rhyacopsyche shorti*, not acicular as in *Rhyacopsyche otarosa*.

#### Description.

*Male*. Length of forewing 2.6–2.7 mm (n=2). Head unmodified, with 3 ocelli; antennae unmodified. Tibial spur count 1, 3, 4. Dorsum of head brown with dark brown setae and light brown patch between antennae; thorax brown with light brown setae dorsally, light brown ventrally; leg segments with dark brown setae. Forewings covered with golden brown setae with small patches of dark brown setae at margins and apex. *Genitalia*. Abdominal sternum VII without mesoventral process. Segment VIII unmodified. Segment IX anterolateral margin very elongate, narrowing, withdrawn into segments VII–VIII, posterolateral margin with rounded setae-bearing dorsolateral lobe and truncate mesolateral lobe. Inferior appendage with rounded mesodorsal projection bearing setae, setae directed anteriad; apex heavily sclerotized, curving dorsad, acute. Tergum X membranous, round in dorsal view, contracted inside dorsolateral lobes of segment IX. Phallus basally tubular, elongate, narrow, apex membranous and with thickened spines.

**Figure 9.**
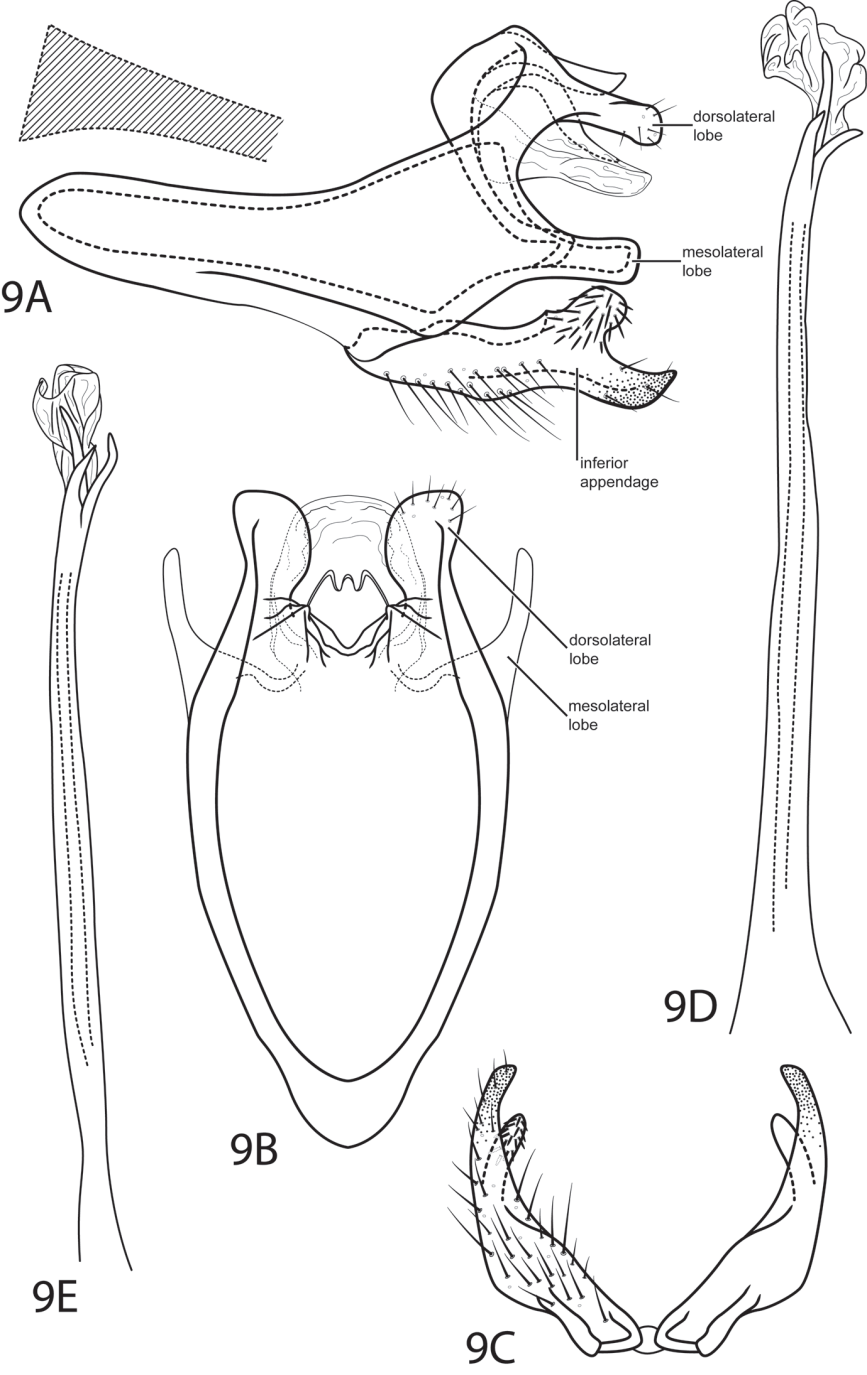
*Rhyacopsyche shorti* sp. n. Male genitalia: **A** segments IX–X, lateral (base of phallus crosshatched) **B** segments IX–X, dorsal **C** inferior appendages, ventral **D** phallus, lateral **E** phallus, dorsal.

#### Material examined.

**Holotype male:** **VENEZUELA: Bolívar:** Gran Sabana, E. Pauji, “Río Curvita”, 04°31.237'N, 61°31.591'W, 869 m, 15–16.vii.2010, UV light, Holzenthal, Thomson, Cressa (UMSP000095199) (UMSP).

#### Paratype.

same data as holotype, 1 male (UMSP).

#### Etymology.

Named in honor of Dr. Andrew Short, an entomologist at the University of Kansas and friend and colleague of the authors.

## Supplementary Material

XML Treatment for Acostatrichia digitata

XML Treatment for Hydroptila cressae

XML Treatment for Metrichia bostrychion

XML Treatment for Neotrichia feolai

XML Treatment for Ochrotrichia spira

XML Treatment for Oxyethira (Tanytrichia) bettyae

XML Treatment for Oxyethira picita

XML Treatment for Oxyethira (Dactylotrichia) quiramae

XML Treatment for Oxyethira redunca

XML Treatment for Rhyacopsyche shorti
